# The anti-scarring effect of corneal stromal stem cell therapy is mediated by transforming growth factor β3

**DOI:** 10.1186/s40662-020-00217-z

**Published:** 2020-11-03

**Authors:** Lin Weng, James L. Funderburgh, Irona Khandaker, Moira L. Geary, Tianbing Yang, Rohan Basu, Martha L. Funderburgh, Yiqin Du, Gary Hin-Fai Yam

**Affiliations:** 1 Department of Ophthalmology, University of Pittsburgh School of medicine, 203 Lothrop Street, Pittsburgh, PA 15213 USA; 2 Shanghai Lanhe Optometry and Ophthalmology Clinic, Shanghai, 200032 People’s Republic of China

**Keywords:** Cornea wound healing, Corneal stromal stem cells, TGFβ3, Inflammation, Fibrosis

## Abstract

**Background:**

Corneal stromal stem cells (CSSC) reduce corneal inflammation, prevent fibrotic scarring, and regenerate transparent stromal tissue in injured corneas. These effects rely on factors produced by CSSC to block the fibrotic gene expression. This study investigated the mechanism of the scar-free regeneration effect.

**Methods:**

Primary human CSSC (hCSSC) from donor corneal rims were cultivated to passage 3 and co-cultured with mouse macrophage RAW264.7 cells induced to M1 pro-inflammatory phenotype by treatment with interferon-γ and lipopolysaccharides, or to M2 anti-inflammatory phenotype by interleukin-4, in a Transwell system. The time-course expression of human transforming growth factor β3 (hTGFβ3) and hTGFβ1 were examined by immunofluorescence and qPCR. TGFβ3 knockdown for > 70% in hCSSC [hCSSC-TGFβ3(si)] was achieved by small interfering RNA transfection. Naïve CSSC and hCSSC-TGFβ3(si) were transplanted in a fibrin gel to mouse corneas, respectively, after wounding by stromal ablation. Corneal clarity and the expression of mouse inflammatory and fibrosis genes were examined.

**Results:**

hTGFβ3 was upregulated by hCSSC when co-cultured with RAW cells under M1 condition. Transplantation of hCSSC to wounded mouse corneas showed significant upregulation of hTGFβ3 at days 1 and 3 post-injury, along with the reduced expression of mouse inflammatory genes (*CD80, C-X-C motif chemokine ligand 5, lipocalin 2, plasminogen activator urokinase receptor, pro-platelet basic protein,* and *secreted phosphoprotein 1*). By day 14, hCSSC treatment significantly reduced the expression of fibrotic and scar tissue genes (*fibronectin*, *hyaluronan synthase 2*, *Secreted protein acidic and cysteine rich, tenascin C, collagen 3a1* and *α-smooth muscle actin*), and the injured corneas remained clear. However, hCSSC-TGFβ3(si) lost these anti-inflammatory and anti-scarring functions, and the wounded corneas showed intense scarring.

**Conclusion:**

This study has demonstrated that the corneal regenerative effect of hCSSC is mediated by TGFβ3, inducing a scar-free tissue response.

## Background

Vision loss is a global burden. Corneal scarring due to trauma or infection is a major cause of blindness with about 10 million people being affected worldwide [1]. The conventional treatment for most existing corneal scars is corneal transplantation (penetrating or lamellar keratoplasty) [2]. Although this procedure is regarded as a highly developed and successful treatment, it is restricted by the global shortage of transplantable donor materials, allograft immune response, long-term graft survival and complications, and the need of surgical expertise [1]. There is a clear need for new approaches to prevent and treat corneal scarring. Corneal stromal stem cells (CSSC), identified in the anterior limbal stroma, represent the mesenchymal progenitors of stromal keratocytes, the dominant cell type inside the corneal stroma [3]. Under our optimized culture condition, primary human CSSC (hCSSC) at early passages exhibit stem cell features, including clonal growth and expression of stem cell markers (CD73, CD90, CD166, SSEA4, OCT4, ABCG2, Nestin and Pax6), as well as the ability to differentiate to keratocytes when induced by specific cytokines, and negligible expression of fibroblast-related genes, such as α-smooth muscle actin (αSMA) and tenascin C (TNC) [4–7]. Using different animal models, including lumican knockout mice and corneal stromal ablation, the applications of hCSSC prevented fibrotic tissue formation, reduced stromal haze, and restored the corneal stromal collagen fibril microstructure and corneal transparency [8–11]. The treated corneas regenerated organized collagenous extracellular matrix (ECM), similar to that in the native corneal stroma [12, 13]. These findings demonstrate the therapeutic potential of hCSSC in repairing stromal damages and regenerating the transparent cornea. The ex vivo propagation allows cells from one donor cornea to be used for multiple recipients or for multiple treatments, thus obviating the shortage of donor materials [14, 15].

The underlying mechanisms of how CSSC reduce corneal fibrosis and scarring, as well as regenerate stromal tissue, have yet to be elucidated. When cultured in low mitogen, ascorbate-containing media, hCSSC expressed an array of genes characteristic of keratocytes [3]. When cultured on a substratum of parallelly aligned nanofibers, the cells deposited layers of collagen fibrils packed in a highly organized pattern, closely resembling the native stromal lamellae [15, 16]. After injection into mouse corneal stroma, the added cells remained viable for many months, and appeared as quiescent keratocytes, expressing typical genes of keratocytes and producing human ECM components [8, 9, 17]. These observations strongly indicate that CSSC differentiate by default into a keratocyte lineage and attain keratocyte functions. Moreover, injection of hCSSC into mouse corneal stroma did not elicit T-cell-mediated immune response, suggesting their ability to immune tolerance and block inflammatory signals [8, 11]. The production of TSG-6 (tumor necrosis factor-stimulated gene 6) in wounded corneas further demonstrated the ability of hCSSC to block neutrophil infiltration, thus reducing the myofibroblast differentiation and scar-associated gene expression, including collagen 3a1 (Col3a1), tenascin C (TNC) and α-smooth muscle actin (αSMA) [18]. Recently, we have shown that hCSSC induced corneal regeneration via an indirect cell-cell interaction, an effect consistent with the paracrine action of extracellular vesicles [19]. Such treatment effects disappeared when the vesicles were depleted of microRNAs, after specific Alix knockdown, suggesting that some microRNAs could have therapeutic effects on corneal fibrosis and stromal regeneration. Whether CSSC promote tissue regeneration by other mechanisms, in particular the scar-free healing response to produce transparent corneas, is under investigation.

Wound healing is regulated by various growth factors, including epidermal growth factor, fibroblast growth factor, platelet-derived growth factor, interleukins (e.g., IL-1, 2, 6, 8) and tumor necrosis factor-α (TNFα) [20–22]. In different organs, including the cornea, members of transforming growth factor β (TGFβ) family are known as key regulators of fibrosis and scar formation; by way of mechanisms underlying the canonical TGFβ/Smad signaling [23–25]. Target inhibition and modulation of TGFβ signaling using small interfering RNAs (siRNA) or neutralizing antibodies to regulate fibrosis and modulate wound healing have been reported in different studies [26–28]. The TGFβ family consists of three closely related isoforms (β1, β2 and β3), with distinct roles in cell differentiation, tissue regeneration and embryonic development [29]. TGFβ1 and β2 mediate tissue fibrosis and scar formation [30, 31], whereas TGFβ3 acts as an inhibitor to these events [32]. The anti-fibrotic and scar-free healing effect of TGFβ3 have been reported in skin, lung and kidney models [33].

In this study, we examined TGFβ3 expression in hCSSC under pro- and anti-inflammatory conditions, mimicking different scenarios of tissue injury and wound healing process. The role of TGFβ3 and its knockdown expression on the stromal regenerative functions of hCSSC were also studied using a mouse corneal wound model.

## Material and methods

### Human corneal biopsies

Human corneal-scleral rims (*n* = 4), from de-identified donors younger than 60 years old and approved for research use, were obtained from the Center for Organ Recovery and Education, Pittsburgh, PA, USA. Tissues used were less than 9 days post-enucleation and preserved in Optisol GS (Bausch & Lomb Inc., Rochester, NY, US). Research followed the tenets of the Declaration of Helsinki and was approved by the University of Pittsburgh Institutional Review Board (IRB) and Committee for Oversight of Research and Clinical Training Involving Decedents (CORID protocol #161).

### Human corneal stromal stem cell isolation and culture

Corneal rims, after the removal of corneal epithelium and endothelium, were dissected to isolate the anterior limbal stroma (1–2 mm wide, 0.5 mm deep), which was then cut into small segments for digestion (16 h at 37 °C) with collagenase A (0.5 mg/ml, Sigma-Aldrich, St Louis, MO, US), as previously described [10]. The single cell suspension was passed through a cell strainer (70 μm pore size, Corning, NY, US) and cells were seeded on culture surface coated with FNC mix (AthenaES, Baltimore, MD, US) with stem cell growth medium (JM-H) containing 2% pooled human serum (Innovative Res, Novi, MI, US) [34]. At 70% confluence, primary cells were collected after brief digestion with TrypLE Express (Thermo Fisher, Waltham, MA, US) and plated at 10 cells/cm^2^ for clonal cell growth in subsequent passages. CSSC from a single donor cornea were expanded to P3 for use in experiments. Cultured cells expressed markers specific for mesenchymal stem cells (MSC) (including CD73, CD90 and CD166), and adult stem cells (including ABCG2, Nestin, Pax6, NGFR) by flow cytometry and RT-PCR [7, 10].

### TGFβ3 knockdown in human CSSC by siRNA transfection

Human CSSC were washed twice to remove dead cells and debris. In fresh culture medium, they were added with a mixture of Viromer Blue transfection reagent for Transfection of Adherent Cells (OriGene, Rockville, MD, US), Viromer Blue buffer and siRNA specific for hTGFβ3 (42 nM; Ambion; antisense sequence: UCA GAG UGU ACA GUC CCA) or scrambled sequences (Silencer™ Select Negative Control #2, Ambion), according to the manufacturer’s protocol. After 24 h, the cells were placed in a Transwell system and co-cultured with RAW at M1 induction (method in next section) for 44 h. Total protein samples were collected for hTGFβ3 and hTGFβ1 expression by western blotting.

### In vitro inflammation assay

In a Transwell system, mouse macrophage RAW264.7 cells (American Type Cell Collection, ATCC, Manassas, VA, US) were plated at 10^4^ cells/cm^2^ in DMEM/F-12 (Thermo Fisher) with 10% fetal bovine serum (FBS, Thermo Fisher) in the lower chamber. They were induced to M1 phenotype by treatment with interferon-γ (IFN-γ, 30 ng/ml; BioLegend, San Diego, CA, US) and lipopolysaccharides (LPS, 100 ng/ml; Sigma-Aldrich) or to M2 phenotype by interleukin-4 (IL-4, 20 ng/ml; BioLegend). hCSSC were seeded at 2 × 10^4^ cells/cm^2^ in the upper chamber and cultured to near confluence before assembly with the lower chamber having RAW cells for co-culture. At regular intervals up to 72 h, RAW cells were collected for the expression of M0, M1 and M2 phenotype markers, while hCSSC were assayed for hTGFβ1 and hTGFβ3 expression.

### Quantitative reverse transcription-polymerase chain reaction (qPCR)

Cells were lysed in RLT reagent (Qiagen, Germantown, MD, US) and total RNA was extracted using RNeasy Miniprep (Qiagen) followed by RNase-free DNase digestion (New England Biolabs, Ipswich, MA, US) according to the manufacturer’s instructions. RNA was precipitated by ethanol and quantified using NanoDrop One (Thermo Fisher). After reverse transcription using SuperScript III RT-PCR kit (Thermo Fisher) and random hexanucleotide primers, cDNA was assayed for candidate gene expression with specific TaqMan probes using universal master mix (Thermo Fisher) or specific target primers (Table 1) using SYBR Green Real-Time Master Mix (Life Technologies, Carlsbad, CA, US) in a StepOnePlus Real-Time PCR System (Applied Biosystems, Foster City, CA, US). Experiments were run in triplicates. The relative RNA abundance was assayed after normalization with housekeeping 18S or glyceraldehyde 3-phosphate dehydrogenase (GAPDH) genes and compared to M0 condition. The fold changes of each gene were calculated by 2^-ΔΔCT^ and expressed as mean ± standard deviation (SD).

**Table 1:** Expression primers

	Gene	GeneBank Accession No.	Sequences
**Oligonucleotide primers**			
1	hTGFβ1	NM_000660.7	F: GACTGCGGATCTCTGTGTCATT
			R: CAGTAGTGTTCCCCACTGGTC
2	hTGFβ3	NM_003239.4	F: CTTCCAGATCCTTCGGCCAG
			R: ATCAAAGGACAGCCACTCGG
3	hTSG6	NM_007115.4	F: AAGGATGGGGATTCAAGGAT
			R: GCTTGTATTTGCCAGACCGT
4	mARG1	NM_031508.1	F: GATTATCGGAGCGCCTTTCT
			R: CCACACTGACTCTTCCATTCTT
5	mCol3a1	NM_009930.2	F: CGTAAGCACTGGTGGACAGA
			R: CGGCTGGAAAGAAGTCTGAG
6	mFN1	NM010233.2	F: TACTCGAGCCCTGAGGATGG
			R: GCAAGGCAACCACACTGACT
7	mHAS2	BC080281	F: CCAAGGTTCTGCTTCCTCAC
			R: CTCTCCATACGGGGAGAGTC
8	miNOS	NM_001313922.1	F: TCACCTTCGAGGGCAGCCGA
			R: TCCGTGGCAAAGCGAGCCAG
9	mSPARC	XM_030245730.1	F: ATGAGGGCCTGGATCTTCTT
			R: CACGGTTTCCTCCTCCACTA
10	mSMA	NM_009696.3	F: TGTGCTGGACTCTGGAGATG
			R: GAAGGAATAGCCACGCTCAG
11	mTNC	NM_035737.2	F: GACTGCCCTGGGAACTGTAA
			R: CATAGCCTTCGAAGCACACA
**TaqMan assay IDs**			
7	hTGFβ1	NM_000660.7	Hs99999918_m1
8	hTGFβ3	NM_003239.4	Hs04398989_m1
9	hGAPDH	NM_001256799.3	Hs01120607_g1
10	mCD80	NM_009855.2	Mm00711660_m1
11	mLcn2	NM_032517.1	Mm01324470_m1
12	mPLAU	NM_032899.1	Mm01149438_m1
13	mPpbp	NM_023785.3	Mm00470163_m1
14	mSpp1	NM_001204203	Mm00436767_m1
15	mG6pdx	NM_032034.3	Mm00656735_g1

*h* = human; *m* = mouse

### Western blotting

Cells were lysed in a radioimmunoprecipitation assay (RIPA) buffer (Thermo Fisher) added with Complete™ protease inhibitor cocktail (Roche, Indianapolis, IN, US) and phenylmethylsulfonylfluoride (PMSF, 1 mM, Sigma-Aldrich). Soluble proteins were denatured in sodium dodecylsulfate (SDS, 2%, Sigma-Aldrich) and β-mercaptoethanol (1%, Sigma-Aldrich), resolved by 4 to 20% SDS-polyacrylamide gel electrophoresis (SDS-PAGE, BioRad, Hercules, CA) and transferred to PVDF-FL membrane (BioRad). After blocking with 5% non-fat milk, the membrane was incubated with antibodies against TGFβ3 (Abcam), TGFβ1 (Abcam) and housekeeping α-tubulin (Sigma-Aldrich), followed by species-specific secondary IRDye-labeled antibodies (LI-COR Biosciences, Lincoln, NE, US). Staining signals were detected and digitized using Odyssey scanner (LI-COR). The specific band density of TGFβ3 was measured and normalized to that of α-tubulin for relative expression in each sample. Experiments were conducted in duplicates.

### Immunocytochemistry

Cells cultured on sterile glass coverslips were fixed in 2% neutral-buffered paraformaldehyde (EM Sciences, Hatfield, MA, US) for 20 min at room temperature. The rinsed samples were permeabilized and blocked in buffer containing 3% normal goat serum (Thermo Fisher) and 0.3% Triton X-100 (Tx, Sigma-Aldrich) for 45 min, followed by incubation with rabbit polyclonal antibody against human TGFβ3 (Abcam) or host species-matched isotype-specific immunoglobulin (Ig) overnight at 4 °C. After washes using PBS with 0.1% BSA and 0.1% Tween-20, the samples were stained with anti-rabbit Ig conjugated with AlexaFluor 488 (Thermo Fisher) and anti-human nuclear antigen-PE (phycoerythrin) conjugate (Abcam) for an hour at room temperature in the dark. After washing, the coverslips were mounted with Fluoromount-G® containing 4′6-diamidino-2-phenylindole (DAPI) (Thermo Fisher) and viewed under confocal microscopy (FluoView 1000, Olympus). Experiments were performed in triplicates.

### Murine corneal stromal wound model and CSSC treatment

The animal experiments were conducted in strict accordance with the Guidelines for the Care and Use of Laboratory Animals of the National Institutes of Health (NIH, Bethesda, MD, US) and The Association for Research in Vision and Ophthalmology statement for the use of animals in ophthalmic and vision research. The protocol was approved by the Institutional Animal Care and Use Committee of The University of Pittsburgh (ISO00012511). C57/BL6 mice (*n* = 20 per experiment, total *n* = 60 for triplicate runs) Charles River Lab, Wilmington, MA, US), 7–8 weeks of age, were housed in an AALAC-approved ABSL2 facility, and provided an unrestricted standard diet. They were anesthetized by intraperitoneal ketamine (50 mg/kg) and xylazine (5 mg/kg), and both eyes received topical proparacaine hydrochloride (0.5%, Alcaine®, Alcon, Fort Worth, TX, US). After saline rinses, the central corneal epithelium (2 mm diameter) was debrided using Algerbrush II (Accutome Inc., Malvern, PA, US) and surgical blade #15. The basement membrane and anterior stromal tissue was further removed by a second application of AlgerBrush [17]. The eyes were rinsed and briefly dried before cell treatment. Immediately after wounding, hCSSC in 10^4^ cells/μl human fibrinogen (70 μg/ml, Sigma-Aldrich) were mixed with 0.5 μl thrombin (100 U/ml; Sigma-Aldrich) and applied to the wound site. After gelation, a second round of fibrinogen and thrombin was applied to the gel surface. The eyes received topical gentamicin (0.3%, Genoptic®, USP, Rockville, MD, US) and ketoprofen (3 mg/kg) for analgesia. We carefully designed the grouping of mouse eyes for injury and cell treatment so that each mouse had one functional eye for daily use and survival. In each set of experiment, 10 mice had one cornea injured, which served as sham control treated with medium without cells; and the other cornea remained normal. Another 10 mice had both corneas injured with one of them treated with hCSSC (reported to retain vision) [9, 10], and the other with hCSSC-TGFβ3(si). Experiments were done in triplicates.

### Corneal scar intensity measurement

At day 14 post-wounding and cell treatment, the eyes were scanned with a Spectral domain Optical Coherent Topography (SDOCT) (Bioptigen, Durham, CA, US). Scanned images were analyzed in a masked fashion regarding the treatments. Image processing and analysis were conducted using ImageJ (NIH) and MetaMorph 7.7.3 (Molecular Devices Inc., San Jose, CA, US) [10]. For scar intensity quantification, pixel intensity were obtained from the corneal region with ImageJ and the average background intensity was subtracted. Control eyes were used to set the lower threshold and scar intensity of each mouse and the overall mean and SD are displayed graphically.

### Mouse corneal tissue collection for RNA expression analysis

At post-wounding and treatment days 1, 3 and 14, mice were sacrificed. After enucleation, corneas were immediately excised on ice. Four to six corneas per group were pooled in ice-cold RLT lysis buffer (Qiagen) and disrupted with a MagNA Lyser Green Beads kit (Roche) at 6000 rpm for 50 s, in a MagNA Lyser Instrument (Roche), ford 6 cycles, each with intermittent cooling. The lysate was passed through QIAshredder column and total RNA was isolated using RNeasy Miniprep (Qiagen) following manufacturer’s instructions. Total RNA (0.5 μg) was transcribed to cDNA by SuperScript III (Thermo Fisher). Gene expression was assayed by qPCR with specific primers or TaqMan probes as listed in Table 1. Experiment was done in triplicates.

### Statistical analysis

All data were presented as mean ± SD. Mean value was compared by two-tailed t tests or ANOVA with a post hoc Bonferroni test using GraphPad Prism 7. Corneal scar intensities were compared using non-parametric Mann-Whitney U test. *P* < 0.05 was considered statistically significant.

## Results

### Human CSSC expressed TGFβ3 in M1 pro-inflammatory phase of mouse macrophages

**Fig. 1:**
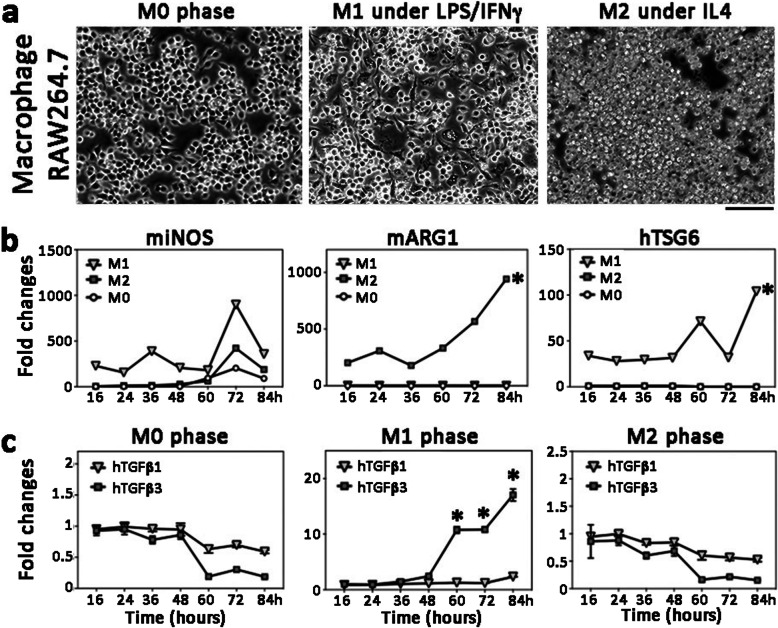
TGFβ3 upregulation in human CSSC under M1 pro-inflammatory condition. **a** Mouse macrophage RAW264.7 at M0 phase was treated with LPS and IFNγ for 48 h to M1 pro-inflammatory phase. The cells were differentiated and became adherent with spindle shape appearance. When treated with IL4, they were induced to M2 proliferating phase mimicking anti-inflammatory condition. Scale bar: 80 μm. **b** M1 and M2 marker expression. Mouse iNOS (miNOS) was upregulated in M1 phase and mouse ARG1 (mARG1) was induced under M2 condition. Co-culture of hCSSC with mouse RAW cells in different phases showed human TSG6 (hTSG6) upregulation under M1 phase. **c** Expression of hTGFβ1 and hTGFβ3 under co-culture of hCSSC with mouse RAW cells in different phases. Significant upregulation of hTGFβ3 in M1, but not in M0 and M2 conditions. Values represent the mean of triplicate experiments. * *P* < 0.05, paired Student’s t-test

The functional response and stability of mouse RAW264.7 macrophages (RAW) was examined by cytokine stimulation to M1 (pro-inflammatory) and M2 (anti-inflammatory) conditions. RAW propagated at M0 phase (Fig. 1a) were treated with LPS and IFNγ to M1 phenotype and showed a consistent upregulation of mouse iNOS (inducible nitric oxide synthase) at various time intervals (Fig. 1b). The M1 macrophages were differentiated, appeared spindle-shape and adhered to the culture surface (Fig. 1a). When treated with IL4, RAW were maintained as small, rounded morphology and had upregulated mouse ARG1 (arginase 1) expression (Fig. 1a and b), indicative of the M2 phenotype. Similar expression patterns were reported in different studies [35, 36]. The co-culture of hCSSC with RAW at the M1 stage demonstrated an inflammation-associated cellular response with an elevated expression of TSG-6 (Fig. 1b). In this pro-inflammatory condition, hTGFβ3 was significantly upregulated (Fig. 1c), starting at 24 h of co-culture and continuing to increase until the end of experiment (84 h). This induction was absent in M0 and M2 phases of macrophages. On the other hand, there was no significant changes of hTGFβ1 expression in hCSSC under M0, M1, or M2 conditions.

**Fig. 2:**
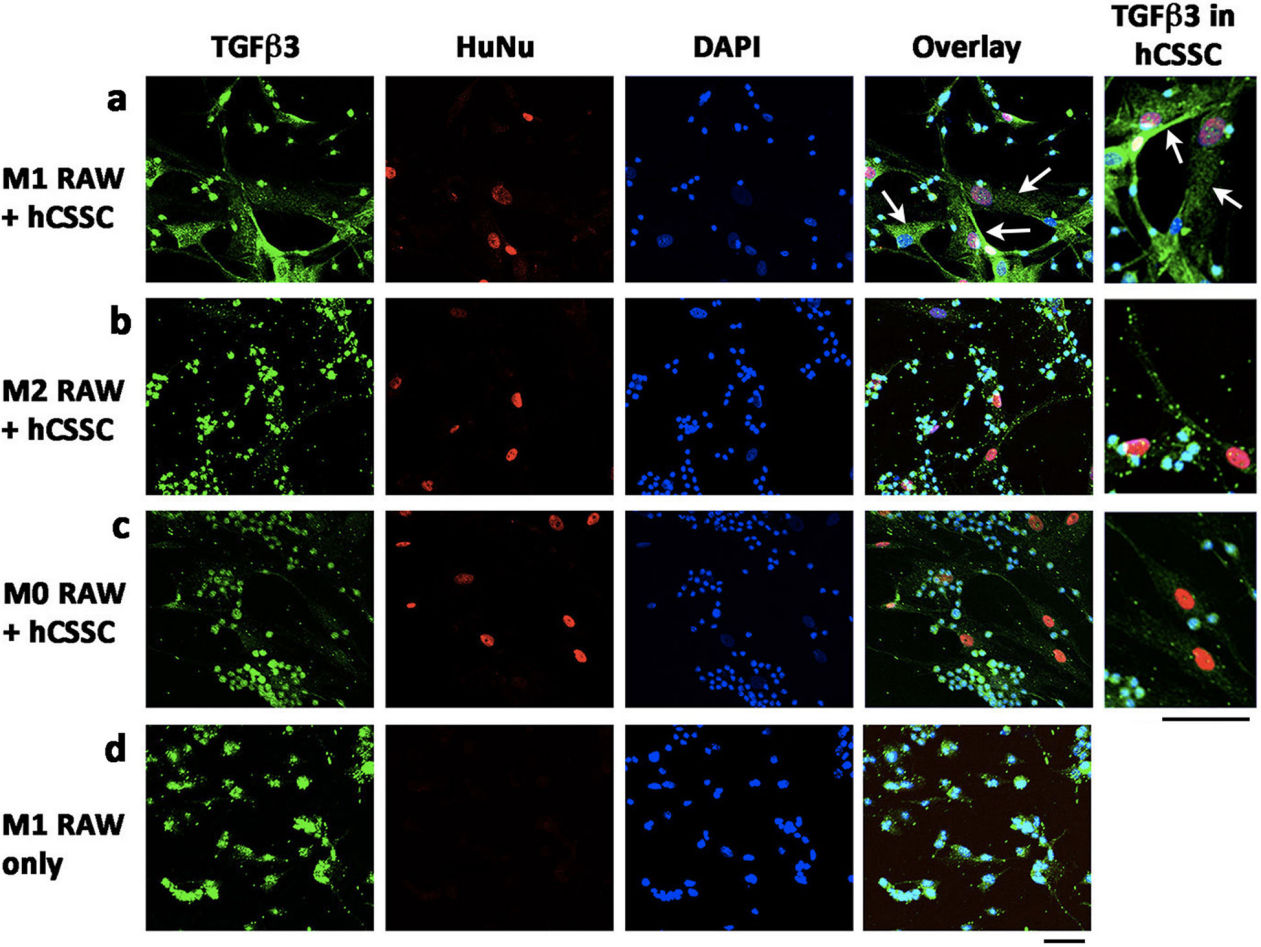
Immunostaining of TGFβ3 expression in hCSSC under co-cultured with mouse RAW cells. After 72 h of co-culture with RAW cells in (**a**) M1, (**b**) M2 or (**c**) M0 condition, hCSSC were distinguished from mouse cells by human specific HuNu labelling (red). A cytoplasmic vesicular-like pattern of TGFβ3 expression (green) was detected in hCSSC (arrow) under M1 condition of RAW cells. The signal was much reduced when co-cultured with RAW cells under M2 and M0 conditions. The immunoreactivity of anti-TGFβ3 antibody was similar in RAW cells under M0, M1, M2 with CSSC co-culture and (**d**) single culture conditions. Experiment was performed in triplicates. Scale bar: 50 μm

Immunostaining showed that TGFβ3 was expressed in hCSSC when co-cultured with RAW cells in M1 phase (Fig. 2a). Since the anti-TGFβ3 antibody appeared labeled in mouse RAW cells (both nuclear and cytoplasmic) (Fig. 2d), the specificity in hCSSC was distinguished by the co-labelling of HuNu (human specific nuclear antigen). In HuNu-labelled hCSSC, TGFβ3 expression was evident under the M1 pro-inflammatory condition. TGFβ3 signals in hCSSC were located inside the cytoplasm as a diffuse punctate pattern with some labelling close to the plasma membrane (Fig. 2a). When co-cultured with RAW cells at M2 and M0 phases, hCSSC negligibly expressed TGFβ3 (Fig. 2b and c).

### M1 macrophages induced hCSSC to produce TGFβ3 via non-contact cell-to-cell communication

**Fig. 3:**
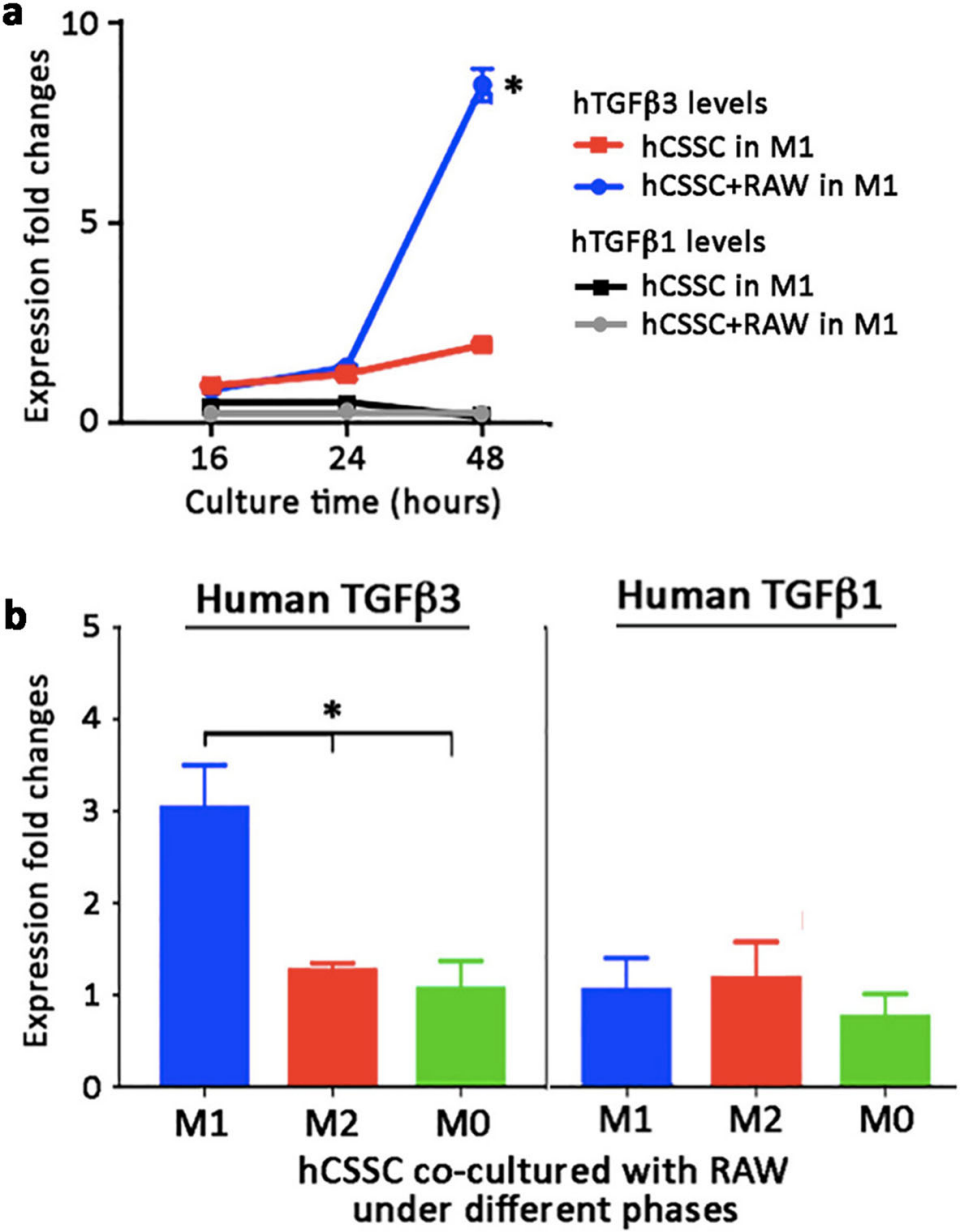
M1-type macrophages induced TGFβ3 expression in hCSSC via paracrine action. **a** In a Transwell system, the co-culture of hCSSC and mRAW cells at M1 condition in separate chambers for 48 h showed a significant hTGFβ3 upregulation from hCSSC. The induction was detectable after 24 h. For hCSSC alone under M1 induction, there were no clear changes of hTGFβ3 expression. The expression of hTGFβ1 was not altered in hCSSC alone or in co-culture with mRAW cells after M1 induction. **b** hTGFβ3 upregulation was significantly greater when hCSSC were co-cultured with mRAW cells under M1 but not under M2 and M0 conditions. hTGFβ1 expression remained unchanged under different conditions. Mean and SD are calculated from triplicate experiments. * *P* < 0.05, paired Student’s t-test

In a Transwell system, hCSSC and RAW cells were placed in separate chambers. In the presence of RAW cells treated with LPS + IFNγ to induce M1 pro-inflammatory condition, the expression of hTGFβ3 in hCSSC was detected after 24 h and was significantly upregulated by 8.2 ± 0.1 folds at 48 h, compared to the untreated control (Fig. 3a). Without RAW co-culture, hCSSC under M1 induction did not show any hTGFβ3 changes. When comparing this Transwell co-culture of hCSSC with RAW cells under M0, M1 or M2 conditions for 36 h, we observed a significant upregulation of hTGFβ3 in hCSSC in the presence of M1 RAW cells, but not with M0 and M2 cells (Fig. 3b). Again, TGFβ1 expression was not altered under different phases (Fig. 3b).

### Suppression of hTGFβ3 expression in hCSSC under M1 condition by siRNA

**Fig. 4:**
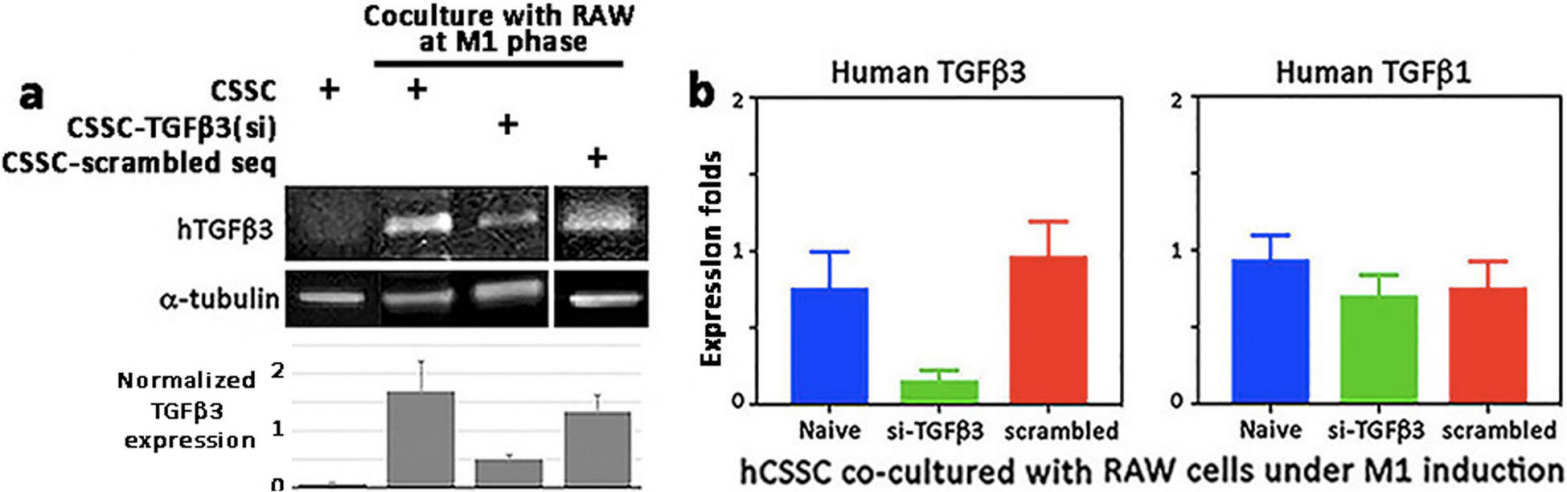
TGFβ3 knockdown in human CSSC. **a** Human CSSC were transfected with hTGFβ3 siRNA or scrambled sequences. After co-culture with RAW cells under M1 phase in a Transwell system for 48 h, naïve hCSSC and hCSSC transfected with scrambled sequences showed similar TGFβ3 upregulation, whereas cells with TGFβ3 siRNA transfection showed reduced expression (decreased by 70–84%) of hTGFβ3. Similar results were obtained in two separate experiments. **b** The reduced hTGFβ3 expression was specific for si-TGFβ3 transfection, while there was no changes for scrambled sequences. The expression of hTGFβ1 remained unchanged with TGFβ3 knockdown in CSSC. Mean and SD are calculated from triplicate experiments

In the Transwell setting, (i) naïve hCSSC, (ii) hCSSC transfected with hTGFβ3 siRNA [hCSSC- TGFβ3(si)], or (iii) hCSSC transfected with scrambled sequences were co-cultured with RAW cells under M1 condition. After 48 h, hCSSC-TGFβ3(si) showed reduced hTGFβ3 expression, by 70–84% of that in naïve hCSSC (Fig. 4a). This was not found for hCSSC transfected with scrambled sequences. The expression of hTGFβ1 was not affected by hTGFβ3 knockdown (Fig. 4b).

### hTGFβ3 was upregulated in mouse stromal wounds after hCSSC treatment

**Fig. 5:**
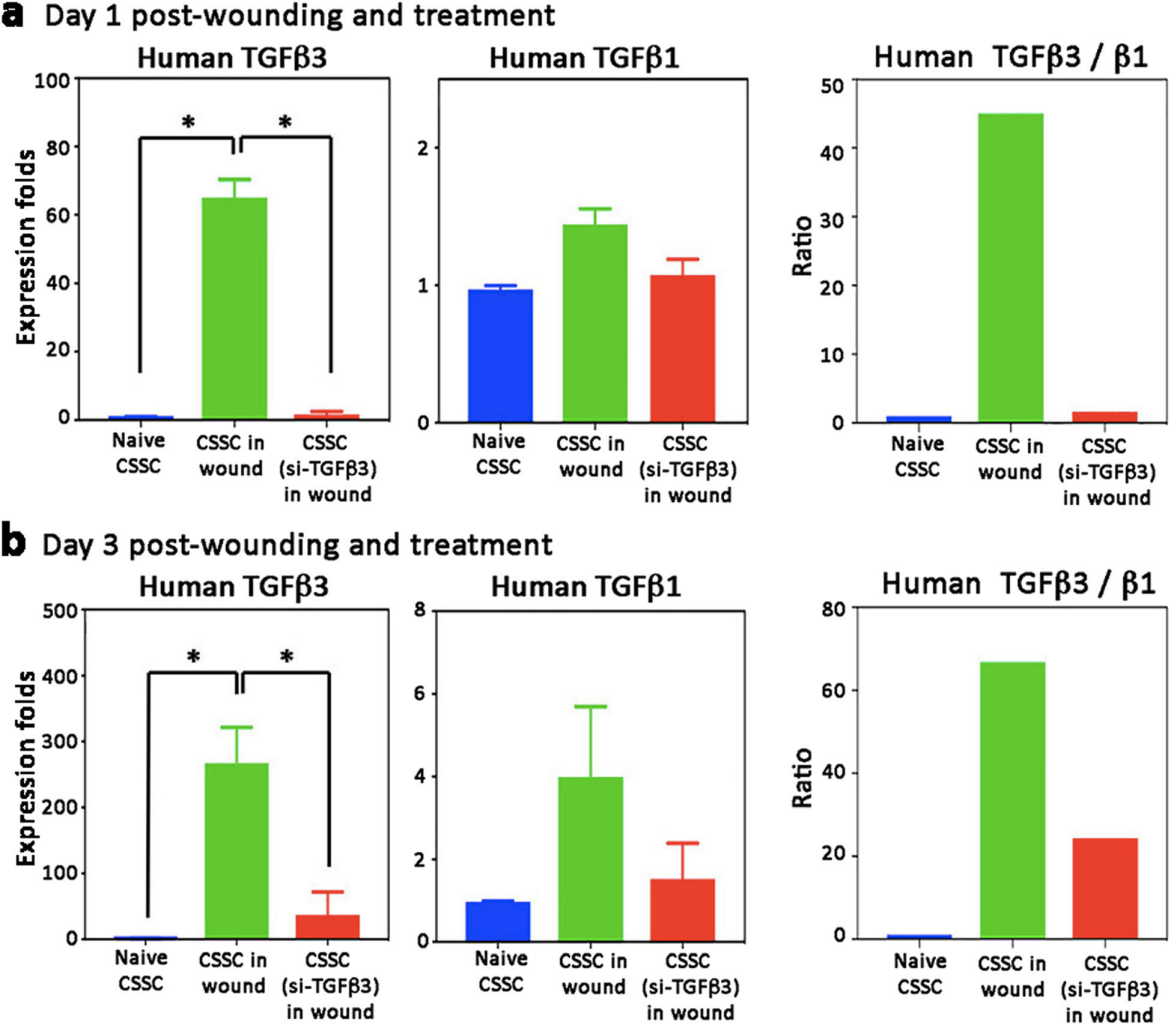
TGFβ3 was upregulated after hCSSC treatment to injured mouse corneas. After stromal ablation wounding, hCSSC or hCSSC-TGFβ3(si), suspended in fibrin gel, were applied to the fresh wounds. At 2 different time points, **a** 1 day and **b** 3 days, the expression of hTGFβ3 was significantly induced in corneas treated with hCSSC but not with hCSSC-TGFβ3(si). On the other hand, the expression of hTGFβ1 was not altered at either time points. The ratio of hTGFβ3/β1 was maintained between day 1 and day 3 post-treatment. Mean and SD represent data from triplicate experiments. *P < 0.05, paired Student’s t-test

Our in vitro models showed hTGFβ3 upregulation in hCSSC under a pro-inflammatory condition. We further explored this association with an in vivo model of corneal injury and wound healing. Human CSSC were applied in a fibrin gel to mouse corneal stromal ablation wounds. At day 1 and 3 after wounding and cell treatment, hTGFβ3 was significantly upregulated, when compared to hCSSC before transplantation (*P* < 0.05, One-way ANOVA) (Fig. 5). The expression of hTGFβ3 on day 1 was increased by 63 ± 8 folds (Fig. 5a) and to a high of 256 ± 63 folds at day 3 (Fig. 5b). This change was negligible with hCSSC-TGFβ3(si) treatment. In contrast, the expression of hTGFβ1 had no change on day 1 post-wounding and treatment (Fig. 5a) and had only mild elevation at day 3 (4 ± 1.8 folds, compared to non-transplanted hCSSC; Fig. 5b). In terms of hTGFβ3/β1 ratio, hCSSC treatment for 1 and 3 days showed similar range of values (Fig. 5a and b). The mean ratio at day 1 was 45 and on day 3 was 65 (*n* = 5 mice per group with injury only and injury with hCSSC treatment, respectively).

### Both anti-inflammatory and anti-fibrotic effects of hCSSC treatment on mouse corneal stromal wounds were reduced by TGFβ3 knockdown

**Fig. 6:**
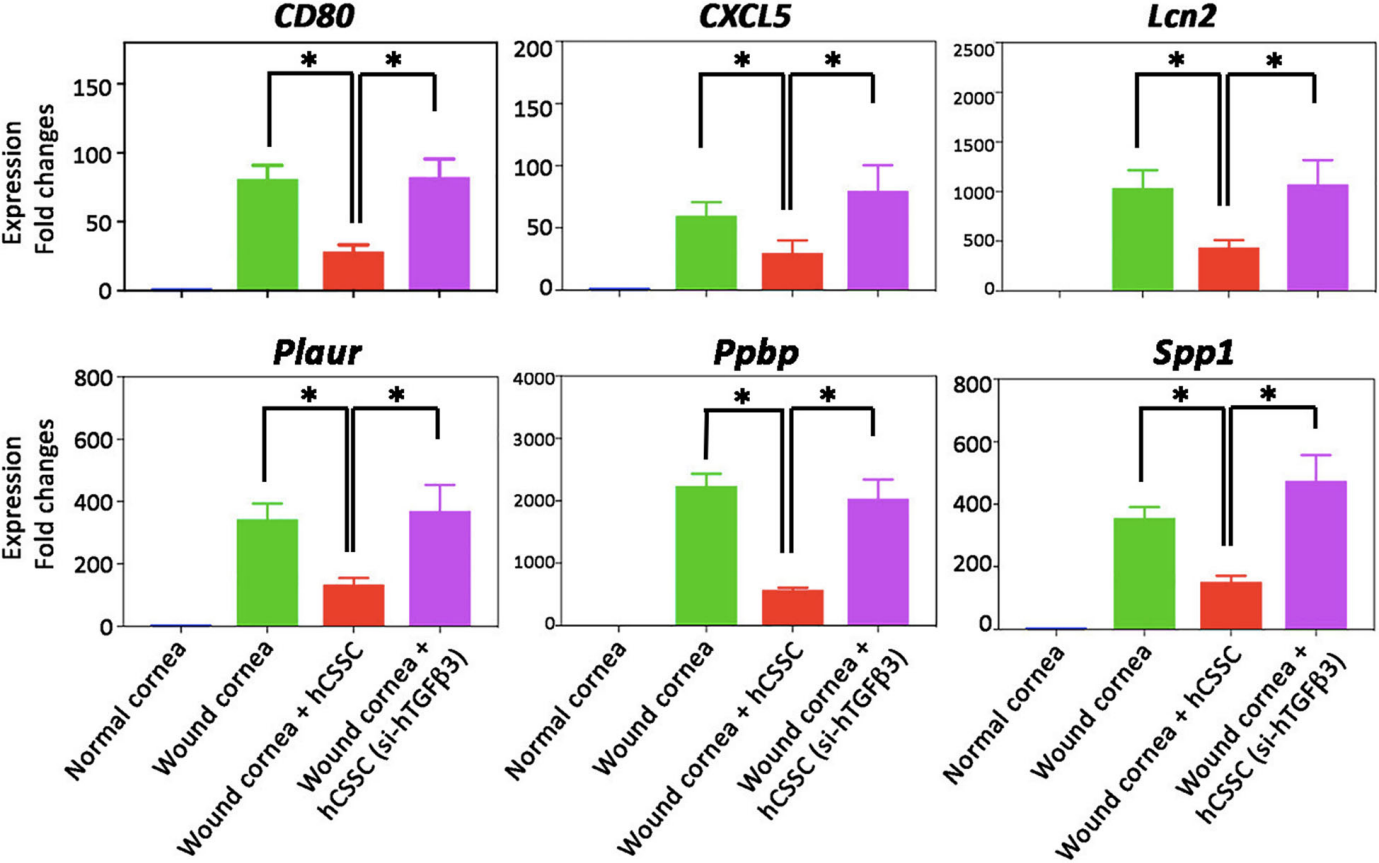
The anti-inflammatory effect of hCSSC on mouse stromal wound model was attenuated by TGFβ3 knockdown. After cell treatment for 1 day, mouse specific early inflammatory genes (*CD80, CXCL5, Lcn2, PLAUR, Ppbp* and *Spp1*) were upregulated in the wounded corneas, as compared to naïve corneas. Gene expression was suppressed by hCSSC treatment, representing an anti-inflammatory effect. Such a response was attenuated when hCSSC-TGFβ3(si) were applied. Mean and SD represent data from triplicate experiments. *P < 0.05, paired Student’s t-test

In our previous analysis using nanoString™ for Mouse Inflammation and Mouse Pan-Cancer Panels, a number of mouse inflammatory genes were affected by hCSSC treatment [19]. We assayed the RNA expression of early inflammatory genes, including *CD80, C-X-C motif chemokine ligand 5 (CXCL5), lipocalin 2 (Lcn2), plasminogen activator urokinase receptor (PLAUR), pro-platelet basic protein (Ppbp)* and *secreted phosphoprotein 1* (*Spp1*). These genes were upregulated at day 1 after wounding when compared to normal corneas, and the elevated expression was significantly suppressed by hCSSC treatment (Fig. 6). This anti-inflammatory effect was abolished by the treatment with hCSSC-TGFβ3(si) (*P* < 0.05, one-way ANOVA), with levels of gene expression similar to that of the wounded corneas without treatment.

**Fig. 7:**
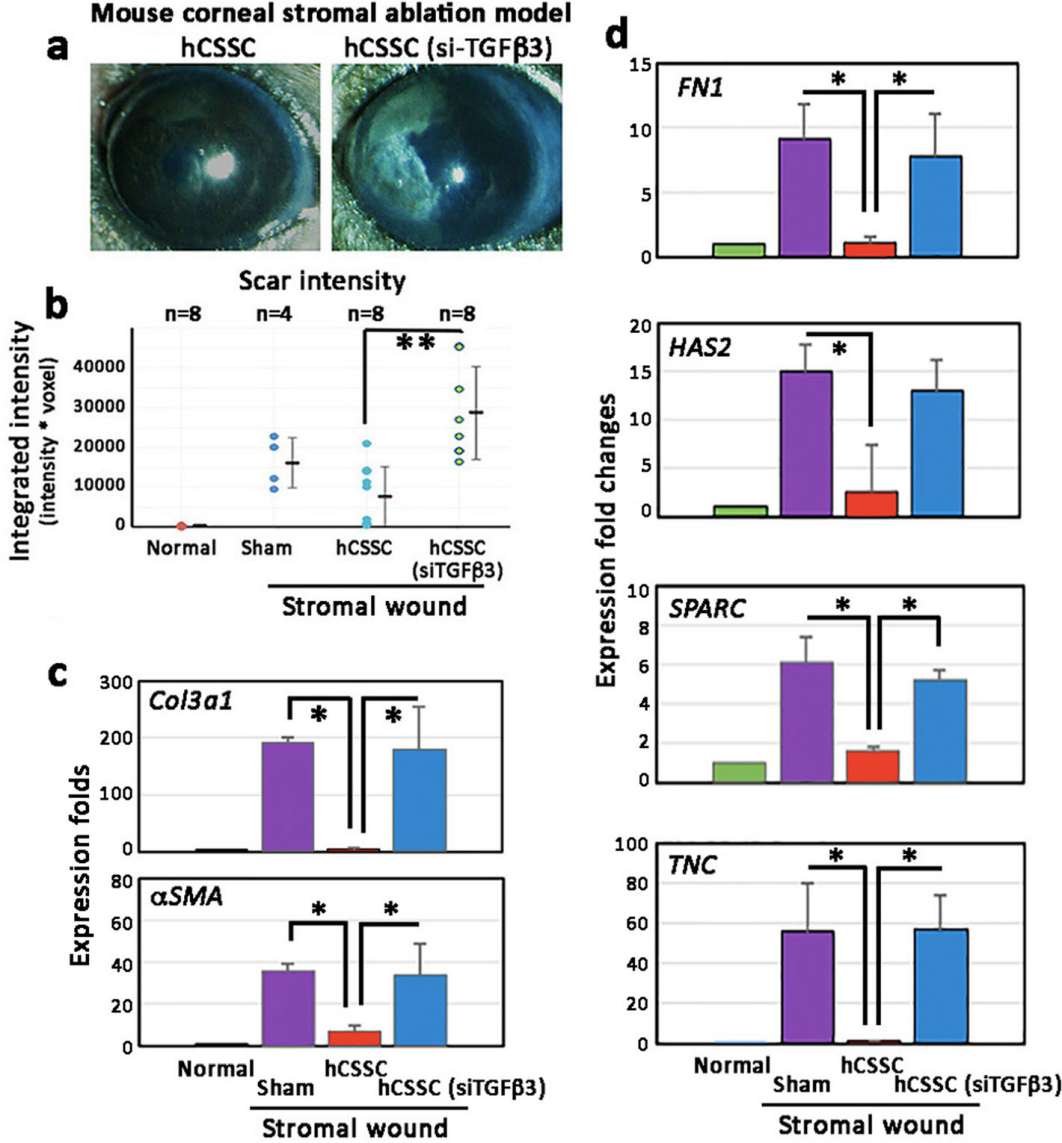
The anti-fibrotic effect of hCSSC on mouse stromal wound model was reduced by TGFβ3 knockdown. Fresh wounds were treated with hCSSC or hCSSC-TGFβ3(si). **a** After 14 days, intense scar was formed in corneas treated with hCSSC-TGFβ3(si), but not with naïve CSSC. **b** Scar intensity measurement showed that corneas treated with hCSSC-TGFβ3(si) had significantly greater scar intensities than those with naïve hCSSC. The levels were similar as in the sham control after wounding. **c** Mouse scar tissue genes (*Col3a1* and *αSMA*) were suppressed after treatment of naïve hCSSC but remained elevated for hCSSC-TGFβ3(si) treatment. **d** Other mouse fibrotic genes (*FN1, HAS2, SPARC* and *TNC*) were also elevated in corneas with hCSSC-TGFβ3(si) treatment, but not with naïve hCSSC. Mean and SD are calculated from triplicate experiments. **b** **P < 0.05, Mann-Whitney U test. **c**, **d** *P < 0.05, paired Student’s t-test

At day 14 post-wounding and cell treatment, the scar-reducing effect by hCSSC was abolished when the treatment used hCSSC-TGFβ3(si) (Fig. 7a). On brightfield illumination of the corneas, the mean scar intensity (*n* = 6 corneas per group) was significantly greater after treatment with hCSSC-TGFβ3(si) when compared to naïve hCSSC (*P* = 0.008, Mann-Whitney U test; Fig. 7b). Expression of mouse fibrosis genes (*Col3a1* and *αSMA*) was significantly suppressed by naïve hCSSC treatment (*P* < 0.05, paired Student’s t-test) but remained elevated with hCSSC-TGFβ3(si) (Fig. 7c). This was similarly observed for other fibrotic genes, including *fibronectin* (*FN1*), *hyaluronan synthase 2* (*HAS2*), *Secreted protein acidic and cysteine rich* (*SPARC*) and *tenascin C* (*TNC*) (Fig. 7d).

## Discussion

In this study, we describe a novel underlying mechanism of hCSSC regeneration of corneal stroma after corneal injury. Specifically, our data demonstrate that: (i) TGFβ3 expression by hCSSC is upregulated in an inflammatory milieu in vitro; (ii) hTGFβ3 is expressed after hCSSC treatment to the stromal wound of mouse corneas and (iii) the specific knockdown of TGFβ3 attenuates these anti-inflammatory and anti-fibrotic activities of hCSSC in vivo. These findings have deepened our understanding on the therapeutic regulation of CSSC to suppress corneal haze and scarring, and to regenerate stromal tissue.

TGFβ3 was expressed by hCSSC in an inflammatory milieu in vitro and in vivo*.* We set up a co-culture system of hCSSC interacting with murine macrophages (RAW264.7 cells) having M0, M1 and M2 phenotypes, mimicking the uncommitted, pro-inflammatory and anti-inflammatory scenarios, respectively, during injury and wound healing process. Human TGFβ3 was significantly upregulated in hCSSC in the presence of M1-type macrophage (pro-inflammatory). The induction was detected at around 24 h of co-culture and escalated thereafter. Similar results were detected when hCSSC were co-cultured with M1 macrophage in a Transwell system, suggesting paracrine action of diffusible molecules from the activated macrophages. Since negligible TGFβ3 changes were detected when hCSSC alone was treated with IFNγ and LPS, the cytokine induction is suggested to be specifically associated to the presence of M1 macrophages, and possibly the secreted molecules from macrophages. The production of IL-1β and IL-6 from activated macrophages has been shown to upregulate TGFβ3 expression in chondrocytes [37, 38]. Here, we show that CSSC might adopt a similar response to the activated macrophages. Further studies will identify the molecular mechanisms underlying CSSC responses in this wound-associated pro-inflammatory condition.

Using an in vivo mouse corneal wounding model, hTGFβ3 expression was also significantly upregulated after hCSSC treatment; there was an increase from day 1 to 3 post-treatment (the mean change of 63 folds at day 1 increased to 256 folds at day 3). At a later time point (day 14), the treated corneas were devoid of haze formation and remained clear, similar to our previous studies [9, 18]. This therapeutic effect was suspended with hCSSC treated to TGFβ3 knockdown. As such, the anti-inflammatory and anti-fibrotic functions were lost, and the injured corneas developed intense scarring. The timing of TGFβ3 production by hCSSC in the early wound healing phase was similar to that of TSG-6 expression, which was also detectable after 1 day of hCSSC treatment on fresh stromal wounds [18]. Corneal injury-induced inflammation is initiated with the infiltration of neutrophils, followed by macrophages. CSSC, displaying the characteristics of MSC, could serve as a guardian against the excessive inflammatory responses through multiple mechanisms e.g., the expression of IL-1 receptor antagonist blocking IL-1 inflammatory signaling, TSG-6 production to reduce NF-κB signaling in the resident macrophages while producing prostaglandin E2 to induce IL-10 expression by macrophages, as well as the expression of sodium dismutase 3 to reduce radical oxygen species in suppressing innate immunity [39]. Among them, the blocking of neutrophil infiltration is crucial at the early wound stage to control inflammation; however, this action might not be effective for anti-fibrosis as hCSSC producing TSG-6 still exhibited fibrotic gene expression [18]. Though our work has demonstrated that CSSC functions through TGFβ3 signaling to control fibrosis development, whether any coordinated or synergistic action by TGFβ3 and TSG-6 in controlling inflammation and wound healing awaits further investigation. In the present corneal wounding study, only female mice were used due to them being less aggressive after wounding and during social housing, to give reliable results. In fact, both sexes have been shown to exhibit similar responses of corneal stromal wound healing, scar formation and stem cell treatment outcomes in our continuous studies (manuscript submitted).

In the human genome, there are 33 functional genes encoding TGFβ family polypeptides [40]. Besides the three TGFβ isoforms (β1, β2 and β3), the family sequences consist of bone morphogenic proteins (BMPs), “growth and differentiation factors” (GDFs), activins, inhibins and nodal proteins [41]. The TGFβ isoforms regulate a variety of cellular processes in both development and adults through gene regulation via Smad- and non-Smad-dependent pathways [42]. In the context of Smad-dependent signaling, which is more extensively studied, the mature and dimeric TGFβ ligands signal through the binding to the cell surface receptor complexes that combine two type I and two type II receptors (TβRI and II), resulting in conformational changes at the ligand-receptor interface and receptor activation [43, 44]. Smad proteins then convey signals from the receptors into nucleus to modulate target gene expression (review in [42]). TGFβ isoforms have pleiotropic functions in controlling physiological phenomena during embryonic development, tissue differentiation and specialization, inflammation and immunity [29]. A broad action profile of TGFβ is also found on the proliferation and differentiation of MSC, production of ECM substances and the chemotaxis effect on various cell types involved in wound healing and the associated inflammatory responses [40].

Among the isoforms, TGFβ1 and β2 is documented to promote fibrosis, fibroblast transition from keratocytes and myofibroblast differentiation, and to induce fibrosis and scar formation after wounding [45]. TGFβ3, on the other hand, is shown to be anti-fibrotic in skin, lung and kidney models [46–48]. In wounded human corneas, an increase in TGFβ3 levels relative to TGF-β1/β2 isoforms was associated with a scar-free phenotype [49, 50]. The presence of TGFβ3 in long-term culture of human stromal fibroblasts resulted in non-fibrotic characteristics and the deposited ECM resembled native stromal tissue. When compared to the treatment with TGFβ1 or β2, the expression of Col3 and αSMA was suppressed by β3, while Col1 synthesis was unaltered. In another study, TGFβ3 reduced the fibrotic gene expression of β1-treated fibroblasts, indicating a possible “rescue” effect in addition to the preventive action on fibrosis development [32]. In a tendon injury model, the addition of TGFβ3 to tenocytes modulated Smad signaling by downregulating Smad3 and its phosphorylation, which suppressed the nuclear translocation of Smad4 and reduced fibrosis-related gene expression [51]. It also upregulated Smad7 to interact with TβR1, which in turn prevented its phosphorylation and activation of receptor-regulated Smad2/3, and thus inhibiting TGFβ/Smad signaling.

Why TGFβ isoforms give rise to different tissue responses is still a mystery. The TGFβ1, β2 and β3 have high sequence homology and share most of their cell surface receptors. After ligand binding, the receptor phosphorylation is important in downstream activities [44]. Additional control can be exerted by ubiquitin and related molecules. TβRs are marked for polyubiquitination by E3 ligases, recruited via Smad7 to TβR1 in complex, for proteasomal degradation, and hence resulting in different signaling responses [52, 53]. Whether TGFβ3 binding to TβR complex stimulates the degradation of a particular receptor type which lead to changes in gene expression is yet to be determined. Moreover, most ligand presentations to their receptors are regulated by co-receptors, which are transmembrane proteins or proteins anchored in the membrane by glycophosphoinositol. They also mediate signaling by other cytokine receptors, giving a broad integration of signal transduction that impacts diverse cellular and pathophysiological conditions. The active TGFβ1 and β3 peptides bind to the TβRII/I complex with high affinity, while β2 engages with this receptor in the presence of co-receptor TβRIII, a cell surface β-glycan [54]. Whether the crosstalk and differential regulation of receptor activities contribute to the different cytokine actions are important topics to be further elucidated.

## Conclusion

hCSSC deliver anti-inflammatory and anti-fibrotic effects via TGFβ3 production, supporting the role of CSSC as a therapeutic agent for corneal repair and regeneration. Without any known harmful effects, exogenous TGFβ3 can be applied to the wound site to stimulate scar-free tissue response; however, CSSC can continuously produce TGFβ3 at physiologically relevant levels. Therefore, the use of native cells is promising in the regeneration of unscarred corneal stroma to restore vision for corneal diseases.

## Data Availability

Data are available from the corresponding author upon reasonable request.

## Abbreviations

ARG1: Arginase 1
Col3a1: Collagen 3a1
FBS: Fetal bovine serum
hCSSC: Human corneal stromal stem cells
HuNu: Human specific nuclear antigen
IL-4: Interleukin-4
iNOS: Inducible nitric oxide synthase
LPS: Lipopolysaccharides
mRAW: Mouse RAW264.7 macrophages
MSC: Mesenchymal stem cells
TGF: Transforming growth factor
TNC: Tenascin C
TNFα: Tumor necrosis factor-α
TSG-6: Tumor necrosis factor-stimulated gene 6
αSMA: α-smooth muscle actin
siRNA: Small interfering RNA
